# Giant Ovarian Tumors in Young Women: Diagnostic and Treatment Challenges—A Report of Two Cases and Narrative Review of the Recent Literature

**DOI:** 10.3390/jcm14041236

**Published:** 2025-02-13

**Authors:** Mariia Melnyk, Andrzej Starczewski, Jolanta Nawrocka-Rutkowska, Amalia Gorzko, Bohdan Melnyk, Iwona Szydłowska

**Affiliations:** 1 Department of Gynecology, Endocrinology and Gynecologic Oncology, Pomeranian Medical University, 70-204 Szczecin, Poland; 2 University Clinical Hospital No. 1, 71-252 Szczecin, Poland; 3 University Clinical Hospital No. 2, 70-111 Szczecin, Poland

**Keywords:** ovarian cysts, ovarian tumor, diagnosis, surgical management, laparotomy, borderline ovarian tumor, serous cystadenoma

## Abstract

**Background:** Ovarian cysts (OCs) are a common gynecological issue, with approximately 20% of women developing at least one pelvic mass during their lifetime. The incidence of large ovarian cysts has decreased substantially due to regular gynecological screenings. However, giant ovarian tumors still continue to pose significant diagnostic and therapeutic challenges. **Methods:** We report two cases of giant ovarian tumors (GOTs). Case 1 involves a 17-year-old woman who presented with a 2-year history of gradual abdominal enlargement, accompanied by repeated attempts at weight reduction. A computed tomography (CT) scan revealed a large tumor. It was excised by laparotomy. Histopathologic examination revealed ovarian cystadenofibroma. Case 2 presents a 25-year-old female who had a 3-month history of progressive, severe abdominal distension and weight gain, accompanied by nausea and diarrhea. CT imaging revealed a giant cystic neoplasm. The cyst was removed by laparotomy. The histopathological study revealed the intestinal–endocervical mucinous borderline tumor. In this context, we performed a narrative literature review, including cases of giant ovarian tumors in young women over the past five years. We centered on diagnoses and management in these cases. **Results:** The surgical management of both cases was successful, with complete tumor excision and favorable postoperative outcomes. hese cases underscore the importance of including giant ovarian tumors in the differential diagnosis of young women presenting with progressive abdominal distension. The narrative review analyzed 39 relevant publications on the management of giant ovarian tumors in young women. **Conclusions:** It is important to highlight a possible risk of malignancy, and risk of fatal complications during the surgical removal of giant ovarian cysts (GOCs). To ensure safer and more successful outcomes, multidisciplinary care should be provided. The early detection and diagnosis of OCs are challenging, as patients may not seek medical attention until the tumor has become large enough to cause symptoms. It is crucial to raise awareness among family doctors and other primary care providers (PCPs) regarding OCs to ensure optimal diagnostic and therapeutic management and improve the outcomes for patients with OCs.

## 1. Introduction

Ovarian cysts (OCs) are a common problem affecting the female population. About 20% of women experience the development of at least one pelvic mass at some point in their lives [1]. These cysts can form at any age but are more frequently observed during the reproductive years and the transition to menarche, which is driven by the body’s natural hormone production. The majority are functional and tend to resolve on their own without medical intervention [2]. OCs were found to have a significant incidence rate of 21.2% among healthy postmenopausal women in Europe [3]. Ovarian tumors represent the most common neoplasms within the reproductive system among adolescents [1,2]. Despite this, they remain infrequently diagnosed. It is estimated that 2.6 out of 100,000 girls yearly are diagnosed with ovarian tumors during childhood and adolescence. Around 10–30% of ovarian masses identified in girls under the age of 17 are malignant, representing 1% of all childhood cancers and 8% of abdominal tumors in children [4,5,6,7,8,9].

Ovarian tumors frequently are asymptomatic and are usually identified during pelvic examination or through ultrasound imaging. Due to widespread access to ultrasonographic technology and the promotion of regular gynecological check-ups, the incidence of huge ovarian cysts has reduced significantly.

The early detection and diagnosis of OCs are challenging, as patients may not seek medical attention until the cyst or tumor has become large enough to cause symptoms. In addition, the first symptoms such as abdominal pain or bloating can have multiple causes, ranging from minor issues to serious medical conditions. In the context of a patient presenting to a family doctor as their first point of contact or other primary care provider (PCP), it can be difficult to pinpoint the underlying cause of the pain.

It is crucial to raise awareness among family doctors and other primary care providers (PCPs) regarding OCs to ensure optimal diagnostic and therapeutic management and improve the outcomes for patients with OCs.

## 2. Detailed Case Descriptions

**Case 1**

A 17-year-old nulliparous, non-smoking, and non-alcohol-drinking female was admitted to the Department of Gynecology, Endocrinology and Gynecological Oncology of Pomeranian Medical University in Szczecin with the suspicion of an ovarian tumor. The patient observed a gradual abdominal enlargement for two years before hospitalization. At first, she sought consultation from a family doctor due to bloating. However, no further diagnostic investigations were conducted. Believing it to be weight gain, she adhered to a calorie-restricted diet and used dietary supplements for weight loss for approximately one year. The patient did not mention any other complaints apart from the abdominal enlargement. She had not experienced any previous illnesses, nor was there any family history of cancer reported.

The patient weighed 54 kg with a 162 cm height and a BMI of 20.5 kg/m^2^. Upon examination, the patient’s blood pressure was found to be 125/85 mmHg, her pulse was 89 beats/min, and her peripheral oxygen saturation (SpO_2_) was 95%. Her vitals were stable.

Upon examination, the abdomen was found to be significantly distended with a palpable mass rising approximately 3 cm above the navel. Transvaginal ultrasound revealed a tumor of 18 cm in diameter.

A complete blood count examination revealed anemia; the hemoglobin level was 10.1 g/dl, and erythrocytes equated to 4.25 mln/uL, with MCV at 73.2 fl; other parameters were normal. Activated Partial Thromboplastin Time (APTT), International Normalized Ratio (INR), sodium, potassium, urea, and serum creatinine levels were found to be within normal ranges. Human epididymal protein 4 (HE4) was increased to 69.1 pmol/L (the normal range (N) was a maximum (max): 60.5 pmol/L). The level of serum lactate dehydrogenase (LDH) was 202 U/L (N max: 48.5 U/L). However, the other tumor markers were found to be within normal ranges: alpha-fetoprotein was 4.54 ng/mL (N max: 5.8 ng/mL), CA-125 was 23.1 U/mL (N max: 35 U/mL), and B-HCG was <0.200 mIU/mL (N < 1 mIU/mL).

**Figure 1:**
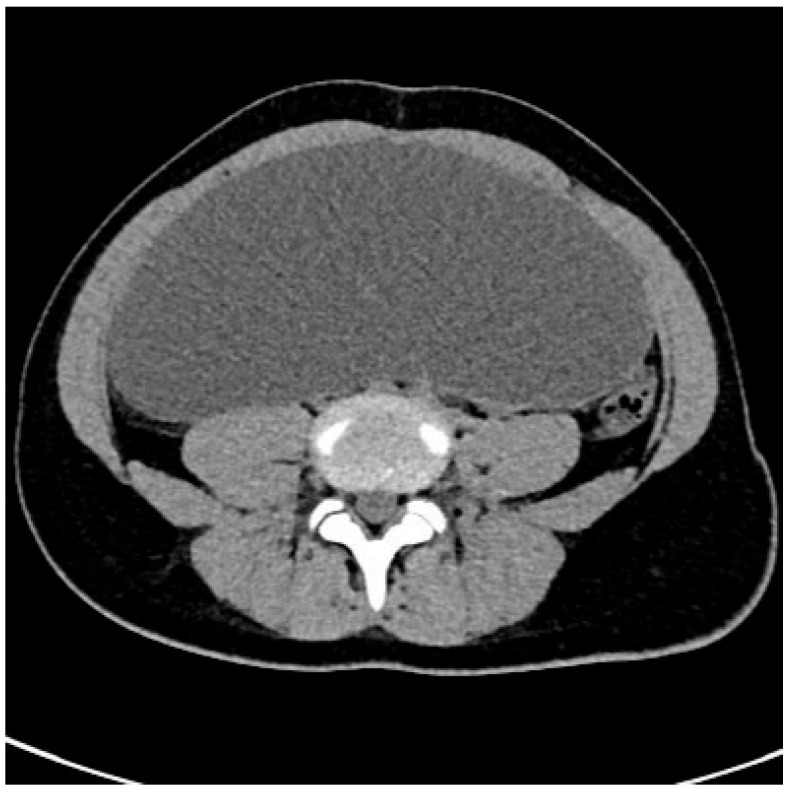
The abdominal CT scan revealed a large tumor in the pelvis and mid-abdomen.

A CT scan of the abdomen and pelvis revealed a large, fluid-filled space with dimensions of 19.3 × 6.2 × 21 cm. The CT scan of the tumor is presented in Figure 1. The lesion was located in the pelvis and mid-abdomen, in front of the uterus, and was compressing the aorta and inferior vena cava. The CT suggested that the lesion probably originated from the left ovary. The walls of the lesion were 1.5 mm thick, and there were no visible partitions or solid elements.

Considering the RCOG guidelines on the “Management of Suspected Ovarian Masses in Premenopausal Women”, which emphasize that there is currently insufficient evidence to recommend a laparoscopic approach and the high risk of cyst rupture and potential dissemination of cancerous cells into the peritoneal cavity during laparoscopy, as well as the need for fertility preservation, an open laparotomy approach was preferred.

A lower midline laparotomy was performed, extending from the umbilicus superiorly to the pubic symphysis inferiorly. After releasing adhesions, a paraovarian tumor measuring 20 cm in diameter was successfully removed without any complications during or after the surgery.

Intraoperative histopathology revealed a serous cystadenoma. Postoperative high-calorie oral nutrition and postoperative rehabilitation physiotherapy were provided. After a period of observation, the patient was discharged without any complications.

The final histopathologic examination revealed ovarian cystadenofibroma.

No complications were observed in the 40 days following the surgery.

**Case 2**

A 25-year-old unmarried nulliparous woman came to our institution in 2023 with a 3-month history of progressive severe abdominal distension and weight gain and the suspicion of ascites. She also reported experiencing nausea and diarrhea. The patient did not have any personal or family history of cancer, significant diseases, smoking, alcohol, or drug use.

Upon admission to the hospital, her height was 160 cm, her weight was 108.5 kg, and her BMI was 42.4. The patient’s blood pressure was 145/98 mmHg, her pulse was 113 beats/min, and her SpO2 was 96% in room air. Her vitals were stable. There was significant abdominal distension, and the skin of the abdomen was thin and with visible stretch marks. Additionally, bowel sounds were diminished.

Upon palpation, a large, firm tumor was felt arising from the lower pelvis, extending to the xiphoid process and symmetrically filling the entire abdominal cavity. The tumor was immobile.

Blood tests showed low MCV, 77.5 fl, and increased lactate dehydrogenase, 292 U/mL. The patient’s renal and liver function parameters (APTT, INR, sodium, potassium, urea, serum creatinine, total protein albumin, and bilirubin levels) were within normal limits. Cancer antigen 125 (CA 125) was 50 U/mL and cancer antigen 19.9 (CA19.9) was 125.0 U/mL. The carcinoembryonic antigen (CEA) was 1.2 ng/mL (N > 3.8 ng/mL), HE4 was 45.9 pmol/L, alpha-fetoprotein was 1.33 IU/mL, and beta-HCG was <0.2 mIU/mL, within the normal range.

**Figure 2:**
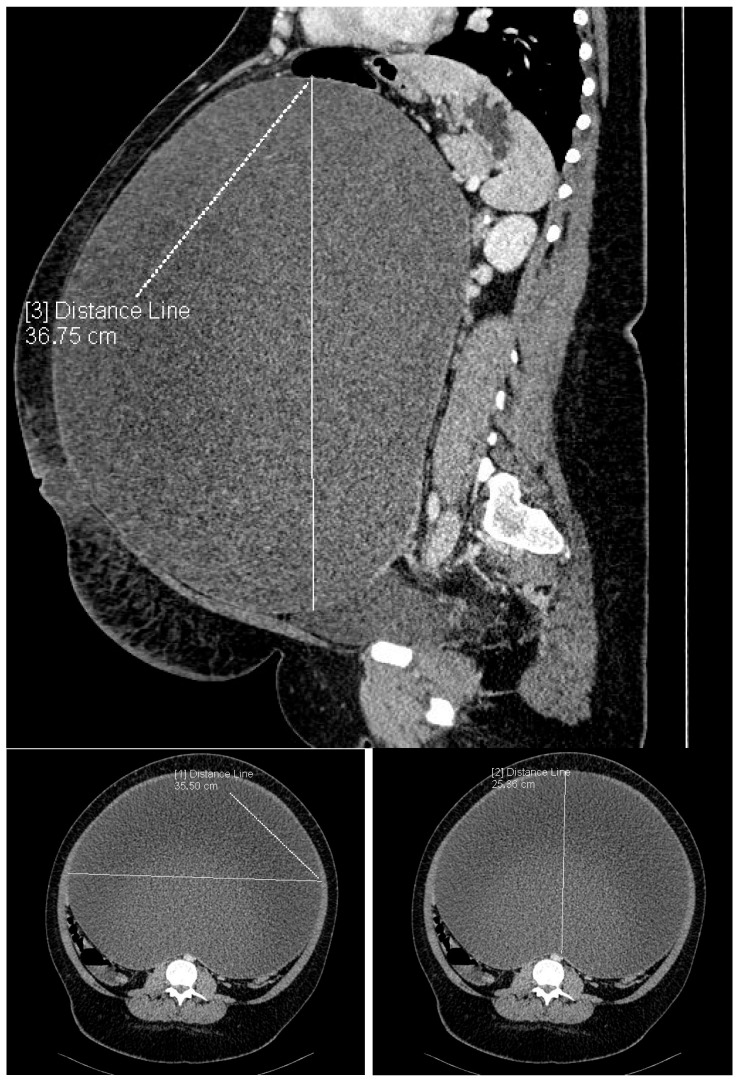
The CT scan of the abdomen and pelvis: measurements of the cystic mass.

A transabdominal ultrasound examination revealed a large cystic lesion of unknown origin occupying the entire abdominal cavity, making it impossible to measure the tumor’s size accurately. As a result, a contrast-enhanced CT scan of the abdomen and pelvis was performed. The CT scan revealed a massive cystic mass with solid components and well-defined margins, measuring 36 × 25.4 × 37.8 cm. The CT scan of the cyst is presented in Figure 2.

The mass was suggestively originating from the left ovary, compressing and pushing the parenchymal organs and intestines dorsally, and pressing on the main vessels. The diameter of the abdominal aorta was 8 mm, and the inferior vena cava was compressed from the confluence of the common iliac veins. Additionally, there were atelectatic bands present in the lower lobes of the lungs, likely due to poorer lung aeration resulting from the high diaphragm setting. A colonoscopy and chest cavity X-ray confirmed no additional findings.

The Risk of Malignancy Index (RMI) was calculated to be 50. Prior to the surgical procedure, the patient’s case was discussed by the Oncological Tumor Board to choose the most appropriate clinical management. Given the higher oncological safety and the need for fertility preservation, laparotomy, tumor resection, and intraoperative histopathological examination were deemed necessary.

The intraoperative histopathological examination revealed the intestinal–endocervical mucinous borderline tumor with a high-grade dysplasia at the part of the tubules in the intestinal component. No signs of infiltrative growth of the tumor were observed.

After obtaining the histopathological results, a resection of the greater omentum was performed. During the surgery, samples were taken from various areas, including the right ovary, visceral peritoneum from the right and left iliac fossa, the sinus of Douglas, and the splenic and hepatic flexures of the transverse colon. The histopathological analysis conducted after the surgery did not reveal any significant pathological changes in those samples.

During the surgical resection of the ovarian tumor, no hemodynamic or cardiac intraoperative complications were observed. The patient did not require any blood transfusions during the procedure. The surgery lasted 205 min, and there were no significant early or late postoperative complications. After the operation, the patient presented with a recurrence of severe wound site pain, necessitating further observation, but no complications were noticed. The patient was discharged from the hospital in good condition and was appointed for follow-up visits to monitor for any recurrence of the tumor.

The final histopathologic examination revealed the intestinal–endocervical mucinous borderline tumor with a high-grade dysplasia at the part of the tubules in the intestinal component.

Following the surgical procedure, the case was reviewed one more time by the Oncological Tumor Board. It was determined that the treatment phase was complete, and patient follow-up was recommended.

Six months after the surgery, the patient was in good health.

## 3. Methods

### Search Strategy

In this narrative literature review, we analyzed available descriptions of cases focused on “giant” and “large” ovarian tumors. A search of PubMed and Medline resources was conducted, aiming at the recent literature, published in the last 5 years (between 2019 and 2024). A combination of the following phrases was used: “large ovarian tumor”, “giant ovarian tumor”, and “young women”, “adolescent”, and “case report”. All retrieved articles (*n* = 847), collected through the electronic search process, were then checked by two researchers. We narrowed our search to articles which presented cases concerned with patients at the age between 10 and 25 years old. Research articles not published in the English language, articles with no abstract available, duplicated articles, correspondence to editors, and summaries of presentations at conferences were all excluded from consideration, as explicitly delineated in the accompanying flowchart.

**Figure 3:**
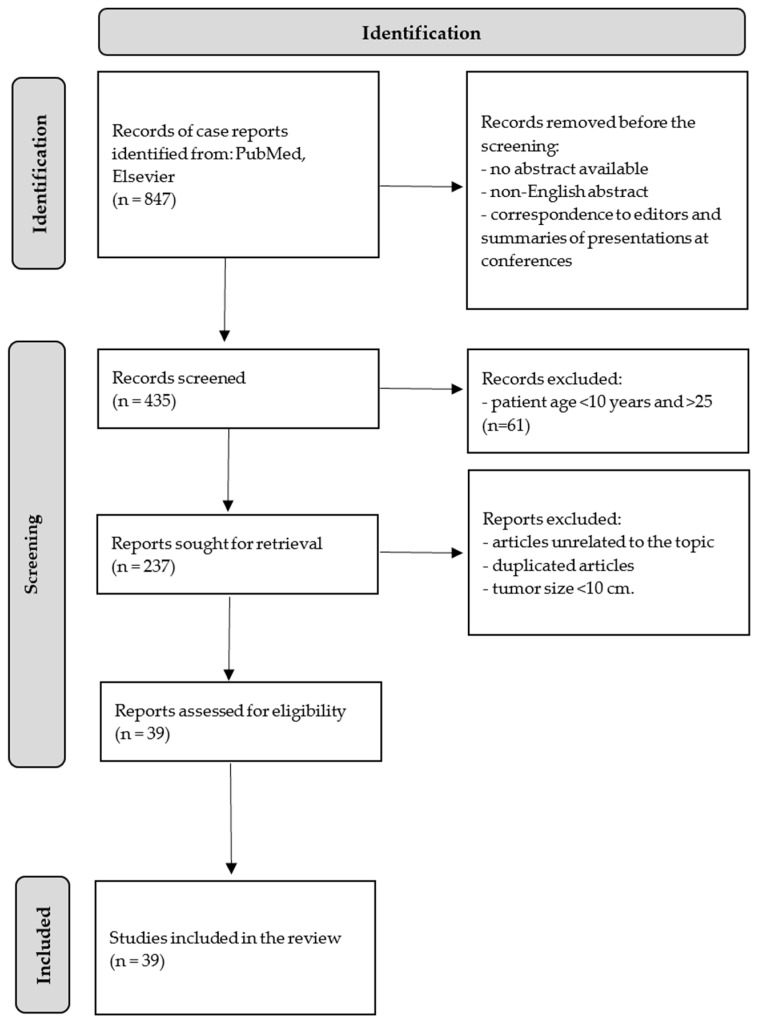
Flow chart of the literature review.

Articles unrelated to the topic and duplicate records found in PubMed were re-moved from the selection process to ensure a focused set of studies. Articles describing cases of ovarian tumors with a diameter of less than 10 cm were excluded from the analysis. After applying all the search criteria, 39 relevant publications were selected for a further analysis (Figure 3). A total of 81 other articles were additionally added to this review.

A comprehensive summary of the clinical data extracted from the narrative literature review is presented in Table 1 for easy reference.

## 4. Discussion and Literature Review

Globally, approximately 7% of women will develop an ovarian cyst during their lifetime. A substantial screening study revealed that the incidence of ovarian cysts among healthy postmenopausal women in Europe is 21.2% [2,49].

Ovarian cysts have the potential to become malignant, and they can also undergo torsion or twisting, leading to pain, bleeding, infection, and even death. Often dubbed the “silent killer”, OCs frequently go undetected in women until they are large enough to be felt externally or have significantly enlarged [50,51]. The clinical symptoms of ovarian cysts typically include progressive enlargement of the abdomen, an indeterminate stomach ache, vaginal bleeding, and symptoms resulting from pressure of the tumor on adjacent organs, such as constipation, early satiety, vomiting, and frequent micturition [52,53,54,55,56,57]. In cases of ovarian cancer, a systematic review conducted by Bankhead et al. determined that 93% of women [95% CI: 92–94%] reported symptoms prior to receiving a diagnosis of ovarian cancer [58]. Findings from case–control studies suggest that these symptoms are notably more prevalent in women diagnosed with ovarian cancer than in those with benign conditions. The sensitivity of single symptoms in detecting ovarian cancer is low [59] but it can be enhanced when these symptoms are presented together. Hamilton and Rossing et al. observed that 85% of women diagnosed with ovarian cancer reported experiencing at least one symptom in the year preceding their diagnosis [60,61].

Notwithstanding, clinicians should show prudence in their assessment, as atypical presentations can occur. For instance, Li X et al. reported a case of a young woman who presented with pleural effusion and ascites, ultimately diagnosed as pseudo-Meigs syndrome. Some authors consider that the pleural effusion could be associated with the movement of ascitic fluid through diaphragmatic pores, the secretion of inflammatory mediators and growth factors, and the blockage of venous or lymphatic drainage caused by a pelvic tumor [33]. In a case documented by Kiemtoré, a woman was complaining that 10 days after a vaginal birth, her abdomen had remained large unlike her previous deliveries. The CT scan revealed the presence of a giant ovarian tumor [46]. A case of huge ovarian mucinous cystadenoma discovered in a patient concomitant with Situs Invertus Totalis was reported by Cu B [41].

The lack of specific symptoms can result in diagnostic errors, as exemplified by a case where a patient presented with a triad of mild abdominal pain, excessive hair growth, and irregular menstrual cycles, reported by Ismail S [35]. After a history of treatment with oral contraceptives to regulate her menstrual cycle, the patient was admitted to a hospital; further investigation ultimately led to the discovery of a large ovarian steroid cell tumor.

However, these most gigantic masses tend to be benign [62]. According to a prospective study involving 1304 females with unilocular cysts who underwent surgery over six years, 93.2% of masses larger than 8 cm in diameter were benign [63].

Currently, there is no universally accepted definition of a “huge” or “giant” ovarian tumor. According to some authors, an ovarian mass is considered large if its diameter ranges from 5 to 15 cm, while a diameter exceeding 20 cm classifies it as giant [64]. In other articles, giant ovarian tumors (GOCs) are defined as those tumors whose size is > 10 cm in diameter [52,62,65,66]. Salem [67] and Sagiv et al. [68] characterized ovarian cysts as “large” or “extremely large” if their upper edge extended beyond the level of the umbilicus. However, since the height of the umbilicus varies among individuals [62], this criterion should be more precise. Regardless of the approach used, it is crucial to establish clear and objective standards for measuring and defining large ovarian cysts.

The exact date of the first report of a patient with a giant ovarian tumor is not something we can provide with certainty. However, giant ovarian tumors have been reported in the medical literature for many years. They are rare but well documented and can present significant challenges in diagnoses and treatment due to their size and potential to cause various complications.

One of the oldest reports of a patient with a giant ovarian tumor dates back to 1905–1906. Spohn et al. reported a case of a 43-year-old female presenting with a simple abdominal cyst. Before surgery, 113 L of gelatinous fluid was drained from the cyst over seven days. The cyst’s total weight was 148.6 kg [69].

The largest ovarian tumor ever recorded had a weight of 137.4 kg and was successfully removed at Stanford Hospital in 1991 by O’Hanlan [70]. However, a more recent case in 2020 involved the successful removal of a 132-pound (about 60 kg) ovarian tumor. The final pathology of the tumor revealed a benign mucinous cystadenoma [71].

When a patient presents with large abdominal masses, it is essential to take into account a broad spectrum of possible diagnoses, including normal physiological intrauterine pregnancy to pathological fibromyomatosis, pelvic endometriosis, urinary retention, abdominal cysts, abdominal pregnancy, intestinal tumors, a hydronephrotic kidney, retroperitoneal tumors, obesity, localized ascites, a urachal cyst, a mesenteric cyst, an abdominal cocoon, or echinococcosis [72].

It is essential to conduct a thorough evaluation and consider all possible diagnoses, and a possible malignant transformation of the tumor, to ensure appropriate management and treatment.

**Figure 4:**
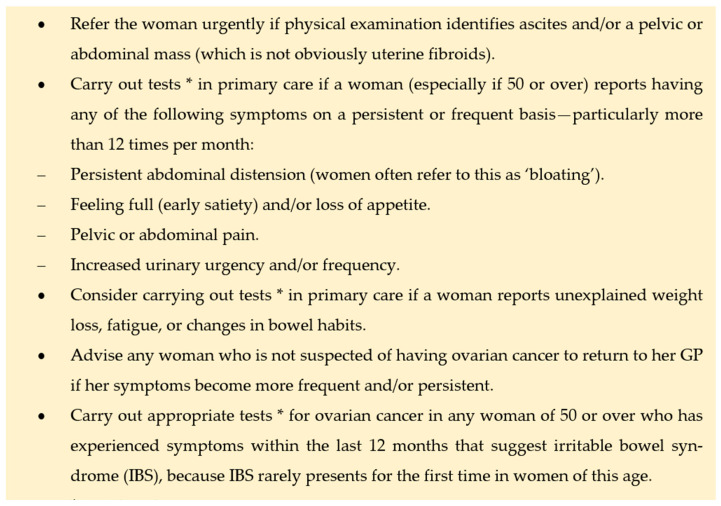
NICE Guidelines for Assessing Potential Ovarian Cancer Symptoms: detection in primary care.

To simplify the diagnostic process for primary care providers, NICE has developed comprehensive Guidelines for Assessing Potential Ovarian Cancer Symptoms (Figure 4).

According to NICE guidelines for the diagnosis of suspected ovarian cancer, it is necessary to measure CA125. In women under 40, alpha AFP and beta-hCG should also be measured. Additionally, an ultrasound of the abdomen and pelvis should be performed. A CT scan of the pelvis and abdomen is recommended. The Risk of Malignancy Index (RMI) should also be calculated [59].

The majority of giant ovarian tumors tend to be benign, while a smaller proportion exhibit malignant behavior. It is important to notice that the statistics are not very precise, as sometimes the study results are contradictory, often due to a small sample size [38,39,40].

Our presented cases included two tumors larger than 20 cm, one of which was a benign cystadenofibroma and the other a mucinous borderline tumor.

Our literature review identified 39 articles on giant ovarian tumors in young women over the past five years. A summary of reviewed cases of giant and large ovarian cysts is presented in Table 1.

In the reviewed cases, histopathological diagnoses revealed six cases of mucinous cystadenoma [23,27,30,31,41,43]; three cases of serous cystadenoma [15,42,46,47]; three cases of ovarian dysgerminoma [17,33,39]; nine cystic teratomas (seven cases presented mature teratomas [12,13,18,26,29,37,45] and two cases presented immature teratoma) [34,44]; three cases of a borderline tumor [10,19,25] (one of them with intraepithelial carcinoma [10]; seromucinous cystadenoma and dermoid cysts [20]); three granulosa cell tumors [16,22,28]; one ovarian steroid cell tumor [35]; primary ovarian leiomyoma [36]; ovarian small cell carcinoma [40]; one yolk sac tumor [24] and one yolk sac tumor associated with minor teratoma lesions [48]; one sclerosing stromal tumor of the ovary [11]; three ovarian mucinous cystadenocarcinomas [14,23,31]; one dysgerminoma-type germinal tumor [32]; and endometrioid ovarian adenocarcinoma [38].

Studies on large ovarian tumors found mature teratomas to be the most prevalent, followed by mucinous and serous cystadenomas [73]. However, another study reported benign teratomas as the most common, followed by serous cystadenomas and endometriotic cysts [74]. Other studies have identified fibromas, seromucinous adenocarcinomas, and low-grade mucinous papillary cystadenocarcinomas [75].

In the study reported by Tjokroprawiro et al., 63 patients with ovarian tumors larger than 20 cm were observed between 2020 and 2022 [75]. A histopathological analysis revealed that 66.67% of giant tumors were malignant, 26.98% were benign, and 6.35% were borderline. Epithelial tumors accounted for 69% of the malignant cases. No significant differences were found in age, tumor size, malignancy, or histopathological type based on the adnexal side. Molecular research categorized epithelial ovarian cancer into subtypes, with type I making up 60.31% and type II making up 14.28%.

To optimally plan treatment for patients with ovarian tumors, we can also assess the risk of ovarian cancer based on ovarian cancer risk factors, which are categorized into two groups: nonmodifiable and modifiable factors. The nonmodifiable risk factors include advancing age [76], genetic predisposition, family history of ovarian cancer [27], personal history of previous cancers, and late menopause. On the other hand, modifiable risk factors comprise nulliparity, hormone replacement therapy (HRT), tobacco smoking, and a diet high in fat. Additionally, several controversial factors have been linked to ovarian cancer, including obesity, exposure to talc powder, radiation exposure, infertility, and the use of fertility medications [77].

Genes with moderate to high penetrance contribute to 5–15% of ovarian cancers. Mutations in BRCA1 carry a 44% risk of developing OC by the age of 80, while that of BRCA2 is 17%; RAD51C and RAD51D result in an 11–12% risk, while those of BRIP1 and PALB2 are 5.8% and 5%. Patients with Lynch syndrome have a risk that ranges from 10% to 15%. Single-nucleotide polymorphisms (SNPs) are associated with a 1.2 to 1.4 times increased risk of epithelial ovarian cancer (OC), while a few may even result in a slight reduction in relative risk (up to 0.8). A significant portion of the remaining 60% of unexplained inherited risk is likely attributed to numerous unidentified low-risk loci, each exerting a minimal effect but collectively contributing to the polygenic risk of ovarian cancer (OC) [78,79,80,81,82,83].

None of these factors were noted in our patients’ history.

Ovarian malignancy is a heterogeneous condition, comprising several biologically distinct subtypes with unique behavioral characteristics [84,85]. These subtypes can be identified and defined based on various criteria, such as the anatomical site of origin, clinical progression, biomolecular tumor profile, and overall genetic instability [86]. Epithelial ovarian cancer is the most prevalent type of the disease. The next ones based on frequency are germ cell and sex-cord-stromal ovarian cancers, which together represent about 5% of all cases [87]. Epithelial ovarian cancer can be classified into four histopathological subtypes: serous (further divided into low-grade and high-grade), endometrioid, clear cell, and mucinous carcinoma [87]. Moreover, malignant epithelial ovarian tumors are often categorized into two broad groups, designated as type I and type II. Type I tumors generally represent the less aggressive clinical subtype, whereas type II tumors are characterized by aggressive progression and are commonly associated with unfavorable survival prognoses [86].

Despite the recognition of distinct ovarian cancer subtypes, current management strategies remain largely uniform and do not adequately address the biomolecular heterogeneity of the disease. Treatment predominantly relies on traditional, non-targeted approaches, except poly(adenosine diphosphateribose) polymerase (PARP) inhibitors in patients with BRCA alterations. Presently, the standard first-line therapy consists of a combination of taxane-based and platinum-based chemotherapeutic agents, applied broadly without the consideration of the underlying clinicopathological and molecular variations within the disease [3].

Ovarian cancer diagnosed at stages III and IV is associated with a poor prognosis and significantly lower survival rates compared to stage I [88]; therefore, a rapid diagnosis of large tumors is crucial. The 5-year survival rate for FIGO stage I ovarian cancer is 90%, stage II is 65%, stage III is 34%, and stage IV is 15% [89]. Some research shows that women diagnosed with OC under 63 have better survival outcomes than those diagnosed at older ages [77]. Survival rates can also vary depending on the specific histological subtype of the disease, as observed by Zhou et al. [90]. For instance, juvenile granulosa cell tumors (JGCTs), despite their high mitotic activity, are typically confined to one ovary and patients with JGCT limited to one ovary generally have a favorable outcome after unilateral salpingo-oophorectomy [22].

For individuals with early-stage, low-grade ovarian cancer (specifically serous, endometrioid, or mucinous expansile subtypes), fertility-preserving surgical approaches have been shown to be a viable and safe treatment option [10,23,91]. This approach may be suitable for patients with stage IC1 tumors, as sub-sequent recurrences in the remaining ovary can usually be successfully addressed through further surgery. However, this strategy is not recommended in stage IC2, and IC3, and grade 3 diseases due to the increased risk of reappearance with a higher likelihood of extraovarian site involvement.

The treatment of giant ovarian tumors (GOTs) generally involves surgical removal of the tumor. While laparoscopic management is currently considered the gold standard for adnexal tumors, many authors consider the tumor dimensions that exceed 8 to 10 cm as a technically challenging factor for this approach [31,32,92,93]. Recent studies suggest that carefully planned laparoscopic cystectomy for large cysts with a low risk of malignancy have shown promising outcomes [21,42,92,94,95,96,97,98,99,100,101,102,103]. However, the RCOG emphasizes that the existing evidence supporting laparoscopy for GOTs remains insufficient [92].

Complete resection without injuring the bowel or other organs along with the prevention of spillage of the cyst fluid into the cavity must be achieved [64,92]. As noted in the literature, surgeons typically perform laparotomy with an “en bloc” resection of the tumors, avoiding the drainage of tumor fluid in most cases [104,105]. Unintentional fluid leakage into the peritoneal cavity is deemed dangerous, specifically in the case of mucinous tumors, because of the risk of developing pseudomyxoma peritonei [52,104]. Chemical peritonitis resulting from the spillage of dermoid cyst contents has been reported in various studies to occur in less than 0.2% of cases [92]. A successful laparoscopic cystectomy case following the preoperative decompression of a tumor using a Veress needle was described by Bhansakarya.

In patients with ovarian tumors who wish to preserve their fertility, conservative treatment like ovarian cystectomy might be preferred [106]. On the other hand, removing a cyst and preserving ovarian tissue can be challenging, but if the cystectomy is not performed meticulously, there is a risk of recurrence. In the case of malignant transformation, complete surgical staging should be performed, and further treatment should follow the ovarian cancer management guidelines [40].

In our specific medical cases, after considering the patients’ history and the size of the mass, a laparotomy was performed, resulting in the complete resection of the tumor without complications in both patients. Because of the patients’ age, fertility-sparing surgeries were performed in both cases.

Operations in patients with such large masses carry a significant risk of potentially life-threatening complications.

In 2007, Yamazume et al. reported a case of death of a patient with GOT 10 h after the operation due to massive abdominal bleeding from the extremely redundant parietal peritoneum, which was a consequence of disseminated intravascular coagulation [107].

Duran et al. documented a case of necrosis in the terminal ileum caused by compression from a massive ovarian cyst that required urgent surgery. Despite the removal of the cyst and right hemicolectomy with side-to-side anastomosis, the patient unfortunately passed away six days later due to cardiopulmonary arrest [108].

Fatal complications reported in the literature include pulmonary and cardiac failure [65], pulmonary embolism [36,109], and sepsis. These cases highlight the challenges and risks associated with the surgical removal of giant ovarian tumors.

The patient’s risk profile for venous thromboembolism (VTE) is elevated by the presence of a large pelvic tumor, which may be exerting mechanical compression on the pelvic vasculature, and is further compounded by the planned major gynecological surgical procedure [36].

One of the potential intraoperative complications associated with the rapid removal of a large tumor is supine hypotension syndrome. This can be effectively prevented through the use of controlled drainage to minimize the risk of hemodynamic instability caused by the sudden decompression of the blood flow to the abdominal gastrointestinal organs located alongside the inferior vena cava [110,111].

Hypovolemia and coagulation abnormalities are frequently observed during the postoperative period. These coagulation abnormalities may be attributed to consumption and dilutional coagulopathy, which necessitates the administration of fresh frozen plasma and platelets to manage the condition [112].

Respiratory complications frequently arise as a result of the abrupt relaxation of the chronically stretched and weakened abdominal and diaphragmatic muscles [113,114]. As a result, delayed extubation is sometimes recommended to prevent respiratory distress and ensure optimal recovery.

Some authors have reported cases of pulmonary edema that can occur after the removal of a large tumor as a result of the abrupt re-expansion of a chronically collapsed lung, which was compressed by the elevated abdomen. To prevent intraoperative and postoperative pulmonary edema, it is recommended to re-expand the collapsed lungs slowly and gradually [115,116].

To minimize hemodynamic instability during surgery, it is essential to perform intraoperative drainage and to settle the patient in the lateral decubitus position. The supine position should be avoided as it can lead to vena cava compression, resulting in a sudden decrease in cardiac output and potentially causing a loss of pulse and cardiac arrest. Therefore, the lateral decubitus position is mandatory to prevent these complications [117,118].

In the presented cases, none of the above-described complications occurred.

Even in instances of benign neoplasms, routine postoperative follow-ups are essential to ensure vigilant monitoring for potential cyst recurrence after surgical excision [58].

To summarize, the intricate nature of ovarian tumors necessitates a coordinated approach to patient care.

The occurrence of large tumors in young women is not uncommon, and there is already some research on this subject. However, it is concerning that, despite improving access to healthcare providers in the 21st century, such cases still occur. Theoretically, such a situation could also be the result of differences in access to healthcare across various locations or social groups. However, no statistics have been found linking giant ovarian tumors to socioeconomic status. In addition, in one of the cases we presented, the patient sought medical help; yet, no diagnostic tests were performed, which highlights a critical issue.

The role of primary care physicians in diagnosing large ovarian masses, as well as ovarian cancer, can be pivotal in expediting the diagnostic process. Deepening the knowledge of primary care physicians facilitates a faster diagnosis and implementation of optimal treatment regimens with a better prognosis for patients [119]. A collaborative effort between medical oncologists, gynecologic oncologists, radiologists, pathologists, and other specialists is essential for crafting tailored treatment plans that cater to each patient’s unique needs. Due to the emergence of new diagnostic and treatment techniques in clinical practice, particularly in oncology, the implementation of multidisciplinary molecular tumor boards (MTBs) could significantly enhance patient care. However, at present, MTBs are available in a limited number of medical centers, which warrants further improvement. Regular multidisciplinary team meetings and ongoing discussions focused on patient experiences facilitate accurate diagnoses and informed treatment decisions, ultimately leading to enhanced patient outcomes.

It is important to highlight that limitations of our narrative literature review are inherent to its design and scope. Firstly, the review was restricted to studies published within the past five years and to the literature available exclusively in English. This may have resulted in the exclusion of relevant studies published in other languages or earlier periods. Additionally, while efforts were made to include a broad range of publications by searching PubMed and Medline, other potential databases were not analyzed, which might have led to the omission of certain pertinent studies. Moreover, as this review focuses on case reports, there is a limitation regarding the generalizability of the findings. The lack of standardization in the presentation of case reports, combined with the varying quality of available descriptions, may have impacted the reliability and comparability of the analyzed data. Furthermore, publication bias may have influenced the dataset, as unusual or atypical cases are more likely to be reported. Finally, the exclusion of cases involving ovarian tumors smaller than 10 cm in diameter, as well as the specific age range of 10 to 25 years, might narrow the scope and applicability of the findings to a less broad clinical context.

## 5. Conclusions

The present case reports emphasize the significance of early diagnostic implementation for ovarian pathologies by primary care providers (PCPs). By showcasing a rare instance of a giant ovarian cyst, this report aims to raise awareness among family doctors, PCPs, and specialized services. An early diagnosis can lead to timely intervention, potentially reducing complications and improving patient outcomes. This report serves as a valuable resource for healthcare professionals, encouraging them to consider ovarian pathologies in their differential diagnoses and seek specialist consultation when necessary.

## Figures and Tables

**Table 1:** Summary of reviewed reported cases of giant and large ovarian cysts.

Main Author	Date of Research	Country of Origin	Age (Years)	Symptoms	Tumor Markers	Ultrasonography	Surgery	Tumor Size (cm)	Histopathological Diagnosis	Complications
Onuzo CN [10]	2024	Ghana	24	Progressively increasing abdominopelvic mass	CA-125—54.3 U/mL	Large cystic abdominal mass arising from the pelvis with echogenic fluid noted with no septations, internal echoes, and papillary excrescences	Right salpingo -oophorectomy	50 cm in the longitudinal dimension	Mucinous borderline tumor with intraepithelial carcinoma	No
Ayyanar P [11]	2024	India	15	Frequent menstrual cycles and heavy menstrual bleeding	CA125—33.5 U/mL, CEA—0.5 ng/mL, CA 19-9—0.80 U/mL, AFP—1.29, LDH—132, β-HCG—1.15 mIU/L	Solid cystic lesion of size 14 × 9.5 cm with increased vascularity; ovaries could not be seen separately	Cystectomy	15 × 14 × 9	Sclerosing stromal tumor of the ovary	No
Grisales-Gallo JE [12]	2024	Colombia	10	Two-day history of intermittent cramping pain in the hypogastrium	-	A mixed mass of 13.6 × 13.8 × 12.7 cm with thick septa in its interior in the left adnexal region	Laparoscopic left oophorectomy	13.6 × 13.8 × 12.7	Mature cystic teratoma and a serous cystadenoma	No
He Y [13]	2024	China	18	Persistent pain in the lower-right abdomen for over 7 h accompanied by nausea and vomiting	-	A 14.1 × 10.1 × 9.0 cm hypoechoic cystic lesion containing a 6.4 × 4.9 × 3.0 cm solid component in the right side of the pelvis, anterior to the uterus	Laparoscopic cystectomy	14 × 13	Cystic teratoma	No
Mbwambo OJ [14]	2024	Tanzania	17	Three-month history of abdominal pain, abdominal distension	-	-	Tumor resection, colectomy, omentectomy, resection of metastasis	30 × 33 × 9	Invasive ovarian mucinous cystadenocarcinoma with metastasis to the colon and omentum	Patient passed away after 6 cycles of chemotherapy
Kluz T. [15]	2023	Poland	23	Increase in abdominal girth, occasional pain on the left side of the abdomen, irregular menstrual cycles, infertility	CA-125, HE4, ROMA, CA-19.9, CA-15.3, AFP, CEA—normal	Massive, fluid-filled tumor mass with segmentally visible solid components; the origin of the neoplasm was not identified	Left salpingo-oophorectomy	80 × 50 × 50	Serous ovarian cystadenoma	No
Park H [16]	2023	USA	12	Secondary amenorrhea, intermittent abdominal pain, galactorrhea	Prolactin—70.5 ng/mL	Large left-sided mass within the pelvis, with concerns for a possible neoplasm	Laparotomy, left salpingo-oophorectomy	15.0 × 9.0 × 18.8	Granulosa cell tumor, juvenile type, FIGO IA	No
Liu S [17]	2023	China	14	Three-month history of abdominal pain	CA125—142.23 KU/L Neuron-specific enolase—148.35 ng/mL β-HCG—5.86 mIU/mL LDH—5204.1 U/L	About 20 cm mixed echo mass in the intraperitoneal and pelvic cavity, mainly solid heterogeneous hypoechoic, scattered in many liquid areas	Transabdominal right ovariectomy + left partial ovariectomy + omentectomy + abdominal para-aortic lymphadenectomy + pelvic lymphadenectomy	19.5 × 6.6 × 15	Bilateral ovarian dysgerminoma	No * Six cycles of chemotherapy after surgery
Barragán-Curiel AE [18]	2023	Mexico	10	Two-month history of increased abdominal girth, distension, and nausea	CA-125: 185.9 U/mL, CEA: 3.28 ng/mL, AFP: 4.67 ng/mL, HE-4: 40.4 pmol/L, ROMA value: 5.4%	Cystic lesion of the ovary and apparent ascites	Cystectomy	20 × 15 × 10	Mature cystic teratoma	No
Yazawa R [19]	2023	Japan	26	-	-	Large multicystic ovarian tumor and massive ascites	Left adnexectomy and appendectomy	Diameter: >20 cm	Mucinous borderline tumor	No * Three courses of chemotherapy
Rodrigues-Martins D [20]	2023	Portugal	23	Progressive abdominal distention	CA-125, CAE, AFP, B-HCG, LDH: normal	A cystic lesion occupying the majority of the abdominal cavity	Bilateral cystectomy	20 × 11 × 27—left ovary, 6 × 8—right ovary	Bilateral dermoid cysts, seromucinous cystadenoma	No
Tzortzopoulou A [21]	2023	Switzerland	15	Persistent abdominal pain	-	Large cystic mass arising from the left ovary	En bloc unilateral oophorectomy and salpingectomy with the use of The Alexis Laparoscopic System	25 × 21 × 21	-	No
Spencer BL [22]	2023	USA	15	Abdominal pain	CA-125 and LDH elevated	-	Left salpingo-oophorectomy with omentectomy	Diameter—22.3 cm	Granulosa cell tumor stage 1C	Six weeks postoperatively: recurrent ovarian cyst
Li Q [23]	2023	China	14	Complaints of a small amount of bloody vaginal discharge	CA-125, HE4, HCG, AFP, CEA, CA19-9: normal	A large, predominantly cystic mass with low internal echogenicity with fine, weakly echogenic spots, containing irregular septations and solid protrusions of varying sizes, resembling a cauliflower	Laparoscopic left salpingo-oophorectomy, appendectomy, omentectomy (single-port surgery)	15 × 9 × 4	Ovarian mucinous cystadenocarcinoma	No
Utami T.W [24]	2023	Indonesia	24	Abdominal enlargement, dyspnea for 3 months	AFP—26.3 ng/mL; CA-125—813 U/mL; LDH, B-hCG: normal	-	A left salphingo-oophorectomy and omentectomy	10.9 × 11.5 × 13	Yolk sac tumor	No * Adjuvant chemotherapy
Gharbia N [25]	2023	Tunisia	30	Four-month history of abdominal pain and a rapid increase in abdominal girth	CA125 = 493 U/mL; CA19–9 = 273 U/ml	An abdominal–pelvic mass of 27 × 12 cm	Right adnexectomy, omentectomy, appendicectomy	30 cm long	Borderline mucinous tumor with signs of infection	No
Kumari S [26]	2023	India	17	Abdominal distention	CEA—12.2 ng/ml	Irregular solid cystic ovarian mass with calcification	Left adnexectomy	15 × 15 cm	Mature terathoma	
Damásio IL [27]	2022	Portugal	20	Rapid and progressive increase in abdominal volume	-	-	Left adnexectomy and cystectomy of the right ovary	38.5 × 31 × 18	Mucinous ovarian cystadenoma	No
Morrison A [28]	2023	USA	14	Intermittent, severe right-lower-quadrant pain, hirsutism, and secondary amenorrhea	Free testosterone—33.6 pg/mL; total testosterone—102.4 ng/dL	A 15 × 10 × 9 cm right, cystic ovarian lesion with possible mural nodularity exerting a mass effect on the right iliac vessels	Laparoscopy followed by exploratory laparotomy with right adnexal detorsion and right oophorectomy	15 × 10 × 9	Granulosa cell tumor	No
AlEssa A [29]	2022	Saudi Arabia	22	One-day history of severe abdominal pain	-	A large ovoid cystic pelvic structure extended into the lower abdomen, measuring 12 × 7 cm. No signs of calcifications or prominent wall blood flow were identified on the color Doppler examination	Unilateral ovarian cystectomy	15 × 10	Mature cystic teratoma with well-differentiated cerebellum	No
Gurung R [30]	2022	Malaysia	25	Three-month worsening abdominal pain and distention	CEA, CA125, CA19-9: normal	-	Right salpingo-oophorectomy	24 × 21 × 12	Mucinous cystadenoma	No
Reham S. [31]	2022	Saudi Arabia	11	Two-year history of lower abdominal pain, severe abdominal distention, increasing abdominal girth	CA-125, HCG, AFP, CEA: normal	A large unilocular cyst arising from the right ovary	Right salpingo-oophorectomy, omentectomy	11 × 20 × 30	Mucinous cystadenocarcinoma	No
Haddout S. [32]	2022	Morocco	18	Abdominal distension, feeling of pelvic heaviness	CA125 = 278 IU/mL, ACE = 1.3 g/L, CA19.9 = 15 IU/mL; HCG—normal	A large solid cystic abdominal mass of an oval shape with clear boundaries measuring 26 × 20 × 10 cm	Left adnexectomy, removal of an infra-gastric peritoneal nodule	20 × 14	Dysgerminoma-type germinal tumor	No * Adjuvant chemotherapy
Li X [33]	2021	China	19	Fever and chest tightness for 2 days	LDH—11,794 U/L CA 125—410.70 U/mL NSE—> 500.00 ng/mL ROMA—27.1%	-	Left oophoro-salpingectomy with radical lymphatic node dissection	29.0 × 20.0 × 13.0	Ovarian dysgerminoma	Pseudo-Meigs syndrome
Małgorzata SŻ [34]	2021	Poland	15	Three-day history of severe pain in the lower abdomen and right lumbar region	AFP—1500 ng/mL, β-hCG > 2 ng/mL	-	Cystectomy	-	Immature teratoma with epithelial elements	Tumor recurrence in 5 months after surgery: mature glial teratoma
Ismail S [35]	2021	Syria	21	One-year history of mild abdominal pain, hirsutism, and oligomenorrhea	-	A sizable mass originating from the left ovary, measuring 154 × 104 mm, exerting pressure on the uterus	Unilateral salpingo-oophorectomy	15 × 10	Ovarian steroid cell tumor	No
Ajayi OA [36]	2021	Nigeria	23	Progressive abdominal swelling of 7 years’ duration	-	A hypoechoic mass in the abdomen occupied the entire abdominal cavity. The uterus and right adnexa were difficult to evaluate, while the left adnexa appeared to be normal	Right ovariectomy and partial salpingectomy	29.5 × 19.5 × 16.5	Primary ovarian leiomyoma	Acute pulmonary embolism
Stavros S [37]	2021	Greece	19	Periodic lower abdominal pain and irritation	-	A 72 × 68 mm mass arising from the left ovary, exhibiting features typical of a dermoid cyst	Laparoscopic ovarian cystectomy	72 × 68	Cystic teratoma with a small hidden ganglioneuroma	No
Tomita S [38]	2021	USA	24	Abdominal bloating	CA-125—235 U/m; CA19-9—49.1 U/mL; CEA—1.0 U/mL	Large, complex, and predominantly cystic mass extending to the level of the abdominal aorta and epigastrum to the pelvis, measuring 23 × 17 × 24 cm	Laparotomy, bilateral adnexectomy	26 × 18.9 × 20.1	Endometrioid ovarian adenocarcinoma	Three cycles of adjuvant chemotherapy
Zhang XW [39]	2020	China	25	Pregnancy (38 weeks 4 days of gestation), lower abdominal pain, and vomiting	CEA, CA-153, SCC: normal B-HCG—14,333 mIU/mL, AFP—142 ng/mL, CA-125—148 U/mL, CA-199—610 U/mL, CA-50—59 U/mL, Cytokeratin 19 fragment—4.86 ng/mL, NSE—76.04 ng/mL	A 23.0 cm by 12.5 cm hypoechoic mass situated at the posterior right side of the uterus; a single live fetus of 38 weeks	Giant tumor and right adnexa resection, omentectomy, appendectomy, pelvic lymphadenectomy, abdominal aortic lymph node biopsy	25 cm × 19 cm × 24	Dysgerminoma of the right adnexa	No
Feng M [40]	2020	China	21	Pregnancy (32 weeks of gestation), abdominal pain, and irregular vaginal bleeding for 5 h	CA125—187 U/mL	-	Hysterectomy, bilateral adnexectomy, pelvic and para-aortic lymph node dissection, appendectomy, partial sigmoid colon resection, omentectomy, peritoneal biopsies	20.5 × 18.3 × 15.5	Ovarian small cell carcinoma of hypercalcemic type, stage III	Intrauterine fetal demise. The patient declined chemotherapy and died 5 months after surgery
Gu B [41]	2020	China	15	Abdominal distension and pain	CA125—84.3 U/mL; CA19-9—326 U/mL	A big mass in the abdominopelvic cavity	Resection of the 2 tumors	28× 25× 20 cm and 15 × 15 × 13 cm	Ovarian mucinous cystadenoma	No
Bhansakarya R [42]	2020	Nepal	25	Abdominal fullness lasting 3–4 months and sensation of mass in the abdomen for 12 days	CA-125, CAE, AFP, LDH: normal	A cystic lesion of 27 × 27 cm in the pelvis	Laparoscopic cystectomy	20 × 18	Serous cystadenoma	No
Leite C [43]	2020	Portugal	20	Abdominal pain and distension, nausea, vomiting, and constipation	CA-125, HE4 CAE, AFP, LDH: normal ROMA—4.1%	A large abdominal cyst of unknown origin	Left adnexectomy	60 cm wide	Ovarian mucinous cystadenoma	No
Brind’Amour A [44]	2020	Canada	16	Abdominal discomfort and distention, and a three-month history of fatigue and anorexia	AFP—7011 ug/L	Large abdominal/pelvic mass with cystic components and microcalcifications	Laparotomy, left salpingo-oophorectomy, left pelvic lymphadenectomy, right ovarian cystectomy, biopsies of the omental and peritoneal implants	23 × 20 × 11	Immature teratoma	Recurrence of the ovarian immature teratoma
Liu YY [45]	2020	China	15	Two-month history of irregular vaginal bleeding, abdominal distension, abdominal pain	CA125—121.20 U/mL, ROMA—39.50%	A large solid–cystic mass in the pelvic cavity, measuring 250 × 195 × 86 mm (solid portion: 52 × 32 mm), with a separation and an anechoic nodule in the wall and punctate blood flow signal	Laparoscopic left salpingo-oophorectomy and separation of pelvic adhesions	19.6 × 12.5 × 26.3	Mature ovarian cystic teratoma containing meningioma and nests of neuroblasts	No
Kiemtoré S [46]	2019	Burkina Faso	25	Gradual increase in the volume of the abdomen during 10 days following vaginal delivery	CA-125—normal	Cystic abdominopelvic image without being able to attach it to an organ	Left total oophorectomy	42 cm long-axis	Serous ovarian cystadenoma	No
Zi Qin Ng [47]	2019	Australia	21	Six-month history of increasing abdominal distention	CEA, CA-19.9, CA-125, chromogranin: normal	Cystic mass measuring 24 × 23 × 14 cm, 22 occupying most of 23 abdomen	Cystectomy	21 × 23 × 14	Serous cystadenoma	No
Stefanelli E [48]	2019	Italy	12	Sudden abdominal pain	AFP—5858 ng/mL; β-hGC and CA 125—normal	A 19 × 13 cm heterogeneous mass with echogenic and hypoechoic features	Left salpingo-oophorectomy	20 × 14 × 9	Yolk sac tumor associated with minor teratomatous lesions	Four cycles of adjuvant chemotherapy

* Additional postoperative treatment.

## Data Availability

Data are available on request, due to the privacy of patients, from the corresponding author.
